# Drug Sensitivity Screening and Targeted Pathway Analysis Reveal a Multi-Driver Proliferative Mechanism and Suggest a Strategy of Combination Targeted Therapy for Colorectal Cancer Cells

**DOI:** 10.3390/molecules24030623

**Published:** 2019-02-11

**Authors:** Jinyan Shen, Li Li, Tao Yang, Niuliang Cheng, Gongqin Sun

**Affiliations:** 1 Department of Biochemistry and Molecular Biology; Shanxi Medical University, Taiyuan 030001, Shanxi, China; shen_jinyan@uri.edu (J.S.); yangtao056cn@126.com (T.Y.); 2 Department of Cell and Molecular Biology, University of Rhode Island, Kingston, RI 02881, USA; lili_5076@126.com; 3 Department of Cell Biology and Medical Genetics, Shanxi Medical University, Taiyuan 030001, Shanxi, China

**Keywords:** colorectal cancer, combination therapy, dose reduction index, protein kinase inhibitors, targeted therapy

## Abstract

Treatment of colorectal cancer mostly relies on traditional therapeutic approaches, such as surgery and chemotherapy. Limited options of targeted therapy for colorectal cancer narrowly focus on blocking cancer-generic targets VEGFR and EGFR. Identifying the oncogenic drivers, understanding their contribution to proliferation, and finding inhibitors to block such drivers are the keys to developing targeted therapy for colorectal cancer. In this study, ten colorectal cancer cell lines were screened against a panel of protein kinase inhibitors blocking key oncogenic signaling pathways. The results show that four of the 10 cell lines did not respond to any kinase inhibitors significantly, the other six were mildly inhibited by AZD-6244, BMS-754807, and/or dasatinib. Mechanistic analyses demonstrate that these inhibitors independently block the MAP kinase pathway, IR/IGF-1R/AKT pathway, and Src kinases, suggesting a multi-driver nature of proliferative signaling in these cells. Most of these cell lines were potently and synergistically inhibited by pair-wise combinations of these drugs. Furthermore, seven of the 10 cell lines were inhibited by the triple combination of AZD-6244/BMS-754807/dasatinib with IC_50_’s between 10 and 84 nM. These results suggest that combination targeted therapy may be an effective strategy against colorectal cancer.

## 1. Introduction

Colorectal cancer (CRC) is the third most commonly diagnosed cancer, and the third leading cause of cancer-related deaths in the US and worldwide [1]. In the US alone, it is projected that medical care cost related to CRC will exceed $17 billion by 2020 [2]. Treatment for CRC relies mostly on traditional options such as surgery, radiation, and chemotherapy [3,4]. Targeted therapy aims to block signaling processes that sustain the proliferation or survival of the cancer cells [5], but for CRC, targeted therapy has so far narrowly focused on blocking the function of EGFR or angiogenesis, mostly using monoclonal antibodies against EGFR or VEGFR [6]. The efficacy of these drugs is limited because many patients are resistant to them. 

Cell proliferation is sustained by signal transduction pathways that relay external growth signals to regulate cellular metabolism and gene expression. Cancer cells often rely on constitutively activated signaling pathways that short-circuit normal signaling and regulatory processes. The most prominent oncogenic signaling proteins are soluble and receptor protein tyrosine kinases (PTKs) [7], molecules in the mitogen activated protein kinase (MAP kinase) pathway [8], and the phosphatidylinositol 3-kinase (PI 3-kinase) pathway [9]. Among the 94 human PTKs, 33 are known to be associated with cancer development, and are referred to as cancer census genes [10]. They include 12 soluble PTKs and 21 receptor PTKs, such as members of the Abl, Src, Jak, PDGFR, EGFR, FGFR, and VEGFR families. Activated receptor PTKs transmit their activation signals by stimulating soluble PTKs, MAP kinase or PI 3-kinase pathways. Constitutive activation of any of these signaling proteins would result in sustained cell proliferation, a hallmark of cancer [11]. The keys to developing targeted therapy are to identify the signaling proteins and pathways that sustain the proliferation of a given cancer, and to block the responsible signaling proteins and pathways [12].

Targeted therapy for CRC has largely relied on monoclonal antibodies against EGFR and VEGFR [3,6]. The autocrine pathway of EGFR is known to be an important factor for carcinogenesis [13], and VEGFRs play central roles in oncogenic angiogenesis for all solid tumors [14,15]. Thus, both targets are not CRC-specific. Furthermore, the most common driver mutations in CRC cells are BRAF V600E mutation and KRAS mutations [16], and both of these mutations would activate the MAP kinase pathway down-stream of EGFR, and inherently render the affected cells resistant to EGFR-targeted therapy [12,17]. Even though many potent and specific inhibitors have been developed against virtually all oncogenic kinases [18], only one small molecule kinase inhibitor for VEGFR, regorafenib (BAY 73-4506), has been approved for CRC targeted therapy [19].

In this study, we evaluate the responses of 10 CRC cell lines to the treatment of a panel of kinase inhibitors. Six of the 10 cell lines are inhibited by multiple inhibitors blocking independent signaling pathways, including the Mek inhibitor AZD-6244, the insulin receptor (IR)/insulin-like growth factor 1 receptor (IGF-1R) inhibitor BMS-754807, and the Src kinase inhibitor dasatinib. Mechanistic analysis and drug combination studies indicate that the proliferation of these cells are supported by multiple pathways, and can only be effectively blocked by combination of drugs that respectively block these pathways. This study provides a mechanistic explanation for why targeted therapy has not worked well for CRC, and a rational strategy for combination targeted therapy. 

## 2. Results

### 2.1. Protein Kinase Inhibitors are Selected to Probe the Oncogenic Signaling of CRC Cell Lines

To determine the responses of CRC cells to signaling inhibitors, we screened the responses of 10 CRC cell lines to a panel of 8 PTK inhibitors, and a Mek kinase inhibitor and a PI 3-kinase inhibitor. The ten drugs and the kinase targets they block with K_d_ values below 20 nM are shown in Table 1. Six of the PTK inhibitors have been tested toward most of the protein kinases in the kinome using the KINOMEscan technology [20] and the data is available through the Library of Integrated Network-based Cellular Signatures (http://lincs.hms.harvard.edu/kinomescan/). Two of the PTK inhibitors, BMS-754807, specific for the kinases in the IR family and Met [21,22], and BGJ398, specific for FGFR family [23], respectively, are also included in this study. Together, the eight PTK inhibitors block most of the cancer census PTKs [10], including members of the Abl, Src, EGFR, FGFR, VEGFR, and PDGFR families. Considering the importance of the MAP kinase pathway [16] and the PI 3-kinase pathway [9] for cancer cell proliferation, a Mek inhibitor, AZD-6244 [24], and a PI 3-kinase inhibitor, AZD-6482 (http://lincs.hms.harvard.edu/kinomescan/), were also included in the current study. 

The CRC cell lines included in this study were randomly selected from ATCC. The genomic sequencing information for all the cell lines is available from the Catalogue of Somatic Mutations in Cancer (https://cancer.sanger.ac.uk/cosmic) [25]. The mutations in BRAF, KRAS, and PIK3CA, and the total numbers of coding mutations in cancer census genes and the whole genomes are shown in Table 2. Each cell line was first screened against all 10 drugs at four concentrations (10 nM, 100 nM, 1 μM and 10 μM). When the proliferation of a cell line was inhibited more than 50% by a drug at 10 μM, the IC_50_ was determined by testing the responses of the cell line to a 2-fold dilution series of 16 concentrations from 20 μM to 0.6 nM.

### 2.2. Some CRC Cells Are Not Sensitive to Kinase Inhibitors

**Figure 1:**
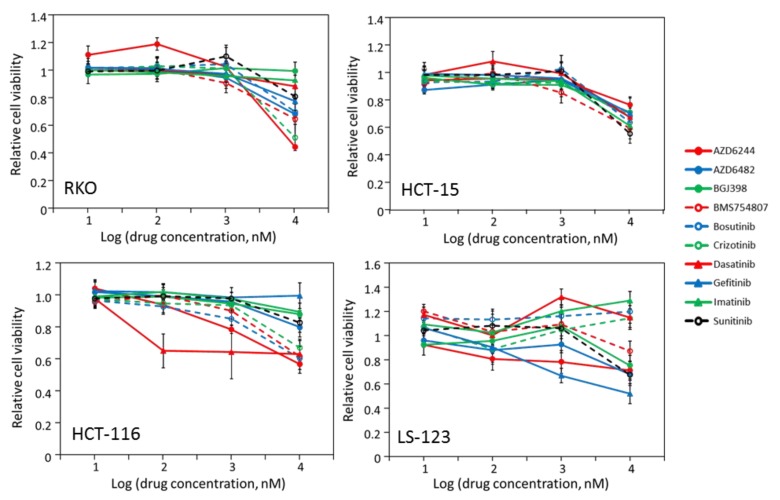
Drug sensitivity of colorectal cancer cells, RKO, HCT-15, HCT-116 and LS-123. Cell viability was determined after the cells were incubated with the drugs at indicated concentrations for 72 h using the MTT assay as described in Materials and Methods. Presented are based on two independent assays containing triplicates. The cell lines are labeled in the graphs. Cell viability incubated in the absence of the inhibitors is taken as 1, and standard deviations are presented as error bars.

**Figure 2:**
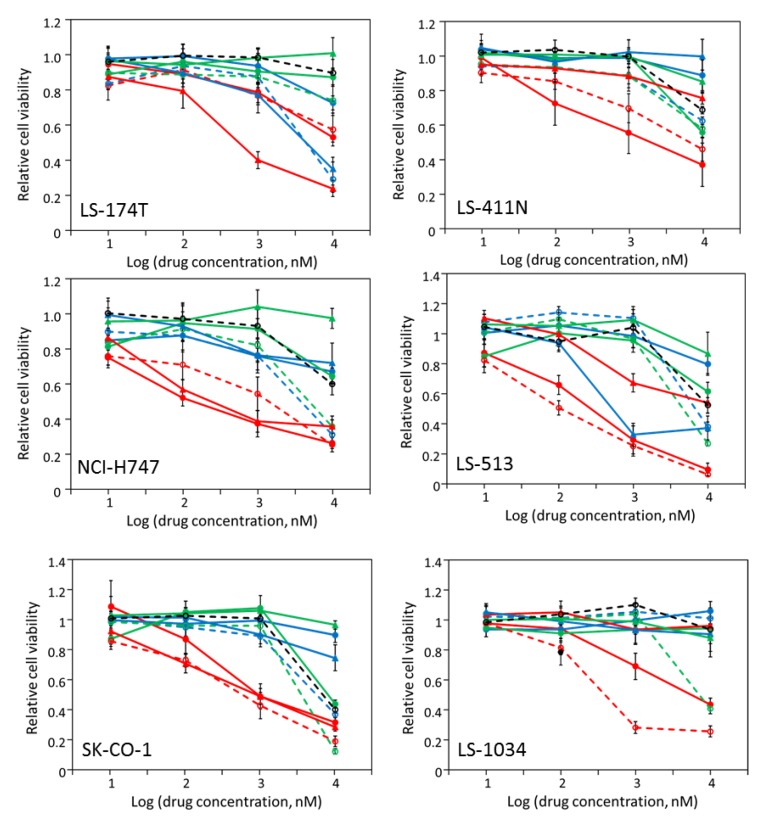
Drug sensitivity of seven colorectal cancer cell lines. The assay and data processing are identical to that of Figure 1.

In the initial four-concentration screen, four cell lines, RKO, HCT-15, HCT-116 and LS-123, were not significantly inhibited by any inhibitors. Figure 1 displays the screening results of these cell lines. For these four cell lines, none of the drugs caused a significant inhibition at 1 μM, or reached 50% inhibition at 10 μM. Considering that these inhibitors block their molecular targets with K_d_/IC_50_ values below 20 nM, the lack of potent inhibition at 10 μM suggests that the target kinases of these drugs do not play major roles in driving the proliferation of these cells. It is noted that three of the four cell lines, RKO, HCT-15 and HCT-116 contain 4300 to more than 11000 coding mutations, 10–20 times more than other cell lines (Table 2). The only non-responder that does not contain a hypermutated genome is LS-123. Despite the fact LS-123 contains a KRAS mutation (G12S), the cell line did not respond to AZD-6244 (Figure 1), or in the 16-concentration assay (data not shown). Two cell lines with hypermutated genomes did respond to at least two drugs: LS-411N to AZD-6244 and BMS-754807, and LS-174T to AZD-6244 and dasatinib (Figure 2).

### 2.3. Most CRC Cells Are Partially Sensitive to Multiple Drugs

Six cell lines responded moderately to multiple kinase inhibitors (Figure 2) and the IC_50_ values for the inhibitory drugs were determined (Table 3). All six cell lines, LS-1034, LS-174T, LS-411N, LS-513, NCI-H747, and SK-CO-1, were inhibited by AZD-6244, with IC_50_’s between 0.28 and 3.01 μM. The sensitivity to AZD-6244, a Mek inhibitor, is consistent with the fact that these cells all contain a BRAF V600E mutation or KRAS mutation(s) (Table 2) that would activate the MAP kinase pathway, thus the down-stream kinase, Mek [26]. Five of the six cell lines, with LS-174T being the only exception, also responded to BMS-754807, a specific inhibitor of IR, IGF-1R, and Met. Because these cells were not at all sensitive to crizotinib (Figure 2), an inhibitor even more potent toward Met [27], the target(s) of BMS-754807 in these cells was most likely IR and/or IGF-1R, even though none of these cells contained any IR or IGF-1R mutations. 

LS-174T, NCI-H747, and SK-CO-1 also responded to dasatinib in addition to AZD-6244, and/or BMS-754807. Dasatinib is a broad spectrum PTK inhibitor, and its most notable targets include Src family kinases, Abl family kinases, and some members of the PDGFR family of receptor PTKs (Table 1) [28]. Because these cells did not respond to imatinib, an Abl family kinase inhibitor, or sunitinib, an inhibitor specific for the PDGFR family of PTKs [29], the most plausible target inhibited by dasatinib is likely Src family kinases. There are eight members in the Src family, but only Src, Yes and Fyn are ubiquitously expressed, with Src being most commonly associated with oncogenic processes. 

### 2.4. AZD-6244, BMS-754807 and Dasatinib Block Independent Signaling Pathways in CRC Cell Lines

**Figure 3:**
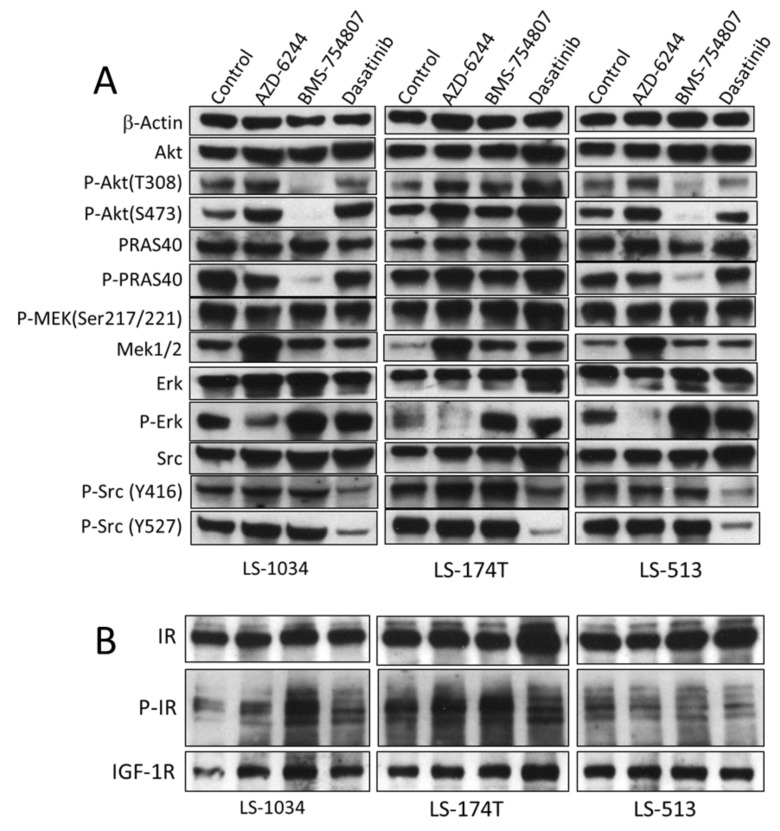
Western blot analysis of the effect of AZD-6244, BMS-754807 and dasatinib on the signaling pathways of selected CRC cell lines. LS-1034, LS-174T, and LS-513 were cultured to 70% confluency, and they were then treated by 5 μM AZD-6244, 2 μM BMS-754807 or 1 μM dasatinib for 1 h. Cells treated with culture medium containing no drug under identical conditions were used as controls. At the end of the treatments, the cells were harvested and lysed directly into the SDS-PAGE sample buffer containing protease inhibitors and phosphatase inhibitors. (**B**), The expression levels and activation status of Mek, Erk, IR, IGF-1R, AKT, PRAS40, and Src were determined by Western blots.

The responses by most of these CRC cells to multiple kinase inhibitors are intriguing. For effective targeted therapy by a single agent, the cancer cell needs to be addicted to a single oncogene or a network [30,31]. Yet it is well established that most cancers gain proliferative advantages because multiple driver genes are activated genetically or epigenetically [32,33,34]. With the CRC cells responding to multiple inhibitors that apparently block independent signaling pathways, we wondered if the complex drug responses were a reflection of the multi-driver proliferative mechanisms. To understand the molecular mechanisms underlying the effects of these kinase inhibitors, we determined how these drugs affected the activation status of several key signaling pathways in selected CRC cells (Figure 3), LS-1034, LS-174T and LS-513.

Even though AZD-6244 is a specific Mek inhibitor, it strongly activated Mek phosphorylation (Ser217/Ser221) in all three cell lines, but inhibited the phosphorylation of Erk (Thr202/Tyr204), a direct substrate for Mek. The fact the AZD-6244 blocked Erk activation and inhibited the proliferation of all three cells indicated that the function of Mek plays an important role for the proliferation of these cells. This is consistent with the fact all these cells contain KRAS mutations. AZD-6244 activation of Mek phosphorylation was somewhat surprising, but it could be a feedback mechanism. Mek phosphorylation on Ser217/Ser221 is catalyzed by Raf, blocking Mek activity by AZD-6244 appears to result in hyper-phosphorylation of Mek, perhaps as an attempt to compensate for the loss of Mek function. This is most likely a feedback mechanism in these cells compensating for the blockade of function by AZD-6244. Such a feedback mechanism has been observed for Akt inhibitor, GSK-690693 in other systems [35]. Nevertheless, these results clearly demonstrate that AZD-6244 exerted its effects on proliferation by blocking Mek activity.

We attempted to determine the protein levels and the phosphorylation status of IR and IGF-1R in all three cells under treatments. While the IR and IGF-1R proteins could be readily determined (Figure 3B), Western blots of the phosphorylated IR and IGF-1R did not reveal a clear result, despite repeated efforts. Invariably, multiple bands were detected by both anti-phospho-IR and anti-phospho-IGF-1R antibodies. Despite the inability to detect the effect of BMS-754807 on IR and LGF-1R phosphorylation level, the effect of BMS-754807 on Akt activation and Akt substrate phosphorylation was reliably clear. In LS-1034 and LS-513 cells, BMS-754807 treatment resulted in significantly decreased phosphorylation of AKT on both Thr308 and on Ser473, suggesting that Akt activation was inhibited in both cells. Consistently, the phosphorylation of the AKT substrate, PRAS40 (Thr246), was also inhibited by BMS-754807 treatment in these two cells. In direct contrast, this was not the case in LS-174T. AKT phosphorylation on both Thr308 and Ser473, and PRAS40 phosphorylation were not inhibited by BMS-754807 in LS-174T, indicating that Akt signaling was not affected by BMS-754807. This lack of AKT inhibition by BMS-754807 is consistent with the fact that the proliferation of LS-174T was also not sensitive to BMS-754807 inhibition. The perfect correlation between AKT inhibition and proliferation inhibition in all three tested cell lines by BMS-754807 demonstrates that the mechanism of action for BMS-754807 is by down regulating Akt signaling. Assuming that BMS-754807 directly inhibited IR/IGF-1R functions, we infer that IR/IGF-1R in LS-1034 and LS-513 mainly stimulate Akt signaling. However, for reasons still unknown, IR/IGF-1R do not activate Akt signaling in LS-174T cells. 

Dasatinib inhibited Src autophosphorylation on Tyr416 and Src phosphorylation on Tyr527, catalyzed by another PTK, Csk, in all three cell lines. This result indicated that dasatinib did block Src kinase activity as well as Csk kinase activity, consistent with its reported specificity. Although normally down-regulation of Tyr527 phosphorylation correlates to activation of Src kinase activity, dasatinib also directly inhibited Src activity, thus the net effect of dasatinib on Src was likely inhibitory. A comparison of the activation levels of Src among the three cell lines suggests that LS-174T contains the highest level of phosphor-Tyr416 in control and under AZD-6244 and BMS-754807 treatments, suggesting that Src activation level was higher in LS-174T than in the other two cell lines. This is consistent with the fact that LS-174T is the only cell line among the three that is sensitive to inhibition by dasatinib. Although phosphor-Tyr416 in LS-1034 and LS-513 was clearly detectable and it was clearly inhibited by desatinib, Src apparently did not play a major role in the proliferation of these two cell lines, as dasatinib did not significantly inhibit the proliferation of these two cells. 

The above results together demonstrate that each of these drugs independently blocks a specific pathway. Thus, they support that hypothesis that these CRC cancer cells are indeed multi-driver/multi-pathway dependent cancer cells. The fact that multiple independent pathways are contributing to the proliferation of these cells explains the observations that these cells are sensitive to multiple inhibitors, but the inhibition by any one of them is only mild.

### 2.5. Most CRC Cells Are Potently and Synergistically Inhibited by the Combination of AZD-6244 and BMS-754807

The results from the previous sections suggested that CRC cell proliferation was driven simultaneously by IR/IGF-1R/AKT pathway, the MAP kinase pathway and/or the Src kinases. One logical prediction of the multi-driver proliferation model is that complete inhibition would require a combination of drugs that collectively block all the activated pathways in a given cell line. We tested this prediction by determining the responses of selected cells to combinations of drugs that were individually only mildly effective. 

**Figure 4:**
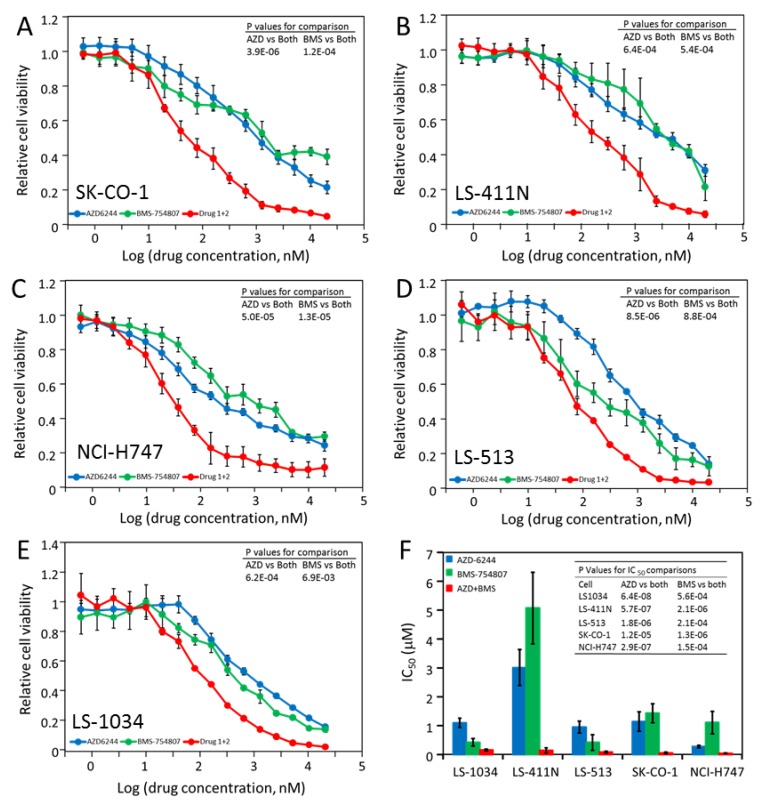
Synergistic inhibition of CRC cell lines by AZD-6244 and BMS-754807. (**A**–**E**) Dose response curves of the indicated cell lines to AZD-6244, BMS-754807 or the combination of the two. Cell viability assay and drug treatments were described in Materials and Methods. The *P* values for the comparisons between the drug combination and each individual drug are shown on the upper right couner. (**F**) Comparison of the IC_50_ values for the individual drugs and the drug combination for all five cell lines. The *P* values for the comparisons in IC_50_ between the drug combination and the individual drugs are shown for each cell line.

As shown in Table 3, five of the ten cell lines (LS-1034, LS-411N, LS-513, NCI-H747, and SK-CO-1) responded to both AZD-6244 and BMS-754807, but the IC_50_’s were mostly in the high nM to low μM range. We determined if these cells would be more effectively inhibited by the combination (1:1 mixture) of the two drugs. As shown in Figure 4A–E, proliferation of each of the cell lines was inhibited by AZD-6244 and BMS-754807, but much more strongly by the combination of the two drugs. The IC_50_’s were summarized visually in Figure 4F. The IC_50_ for the two-drug combination ranged from 46 nM for NCI-H747 to 153 nM for LS-1034. These nM level IC_50_ values indicate that these CRC cell lines are extremely sensitive to the combination of AZD-6244 and BMS-754807. 

**Figure 5:**
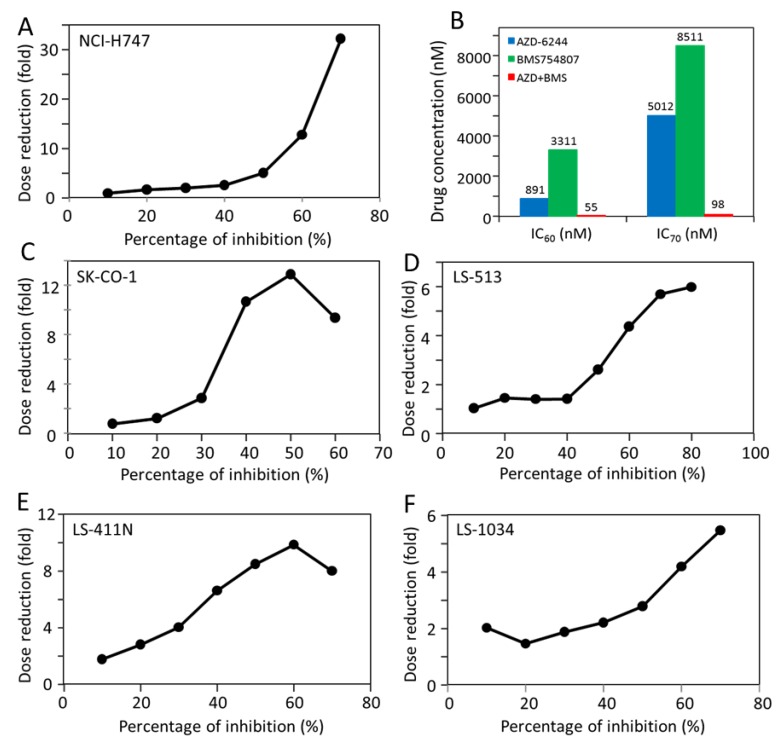
Correlation between the combination synergy and the percentage of inhibition. (**A**,**C**–**F**) Dose reduction index for the AZD-6244 and BMS-754807 combination as a function of the percentage of inhibition in indicated cell lines. The dose reduction indexes were calculated as described in Materials and Methods using the data presented in Figure 4B, IC_60_ and IC_70_ of NCI-H747 for AZD-6244, BMS-754807 and the combination of the two drugs. The dose reduction indexes, the IC_60_ and IC_70_ values reported in these graphs are derived from the data presented in Figure 4. Because statistical analysis was performed in Figure 4, no additional statistical analysis was performed here.

An interesting and potentially very useful characteristic of the cell responses to the drug combination is that the synergy is most striking at higher levels of inhibition. This is best illustrated by graphs of dose reduction index (DRI) as a function of percentage of inhibition (Figure 5). Synergy in drug combination is often expressed as either the combination index (CI) or DRI, two inversely related measures. The CI is a measure of the synergy between two drugs, with lower values corresponding to higher synergy, while DRI is a measure of how many folds the drug doses may be reduced for a given inhibition level, in combination compared with the doses of each drug alone [36,37]. As shown in Figure 5, DRI usually starts around 1 at 10% inhibition level, and increases dramatically as the level of inhibition increases. For example, NCI-H747 has a DRI of approximately 1 at 10% inhibition, and it gradually increases to over 30 at 70% inhibition. This means that the combination is greater than 30 times more effective in achieving 70% inhibition than treatments by the two drugs if there was no synergy between them. The dramatic synergy is also obvious from a comparison of the IC_60_ and IC_70_ values (Figure 5B) for the drugs alone and for the drug combination for NCI-H747. The IC_60_’s are 891 nM for AZD-6244 and 3311 nM for BMS-754807, but only 55 nM for the drug combination. The difference is even more dramatic for the IC_70_’s, at 5012 nM for AZD-6244, 8511 nM for BMS-754807, but only 98 nM for the drug combination. Inhibition of 80% was not achieved by either drug alone up to 20 μM, but achieved by approximately 300 nM of the drug combination. This positive correlation between the level of synergy and the level of inhibition in combination treatments would be a very desirable feature if it is extended to combination cancer therapy. It is a common feature of all five cell lines shown in Figure 5, even though the DRI’s are more dramatic in some cells than in others. Nonetheless, the synergistic benefits at higher inhibition levels are clear in all five cell lines. 

### 2.6. LS-174T Cells Are Sensitive to Inhibition by the Combination of AZD-6244 and Dasatinib

**Figure 6:**
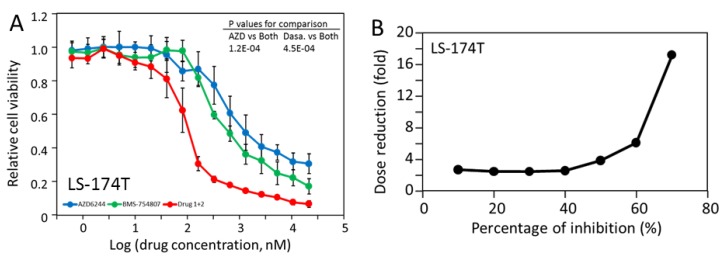
Drug sensitivity of LS-174T. (**A**) The viability of LS-174T in response to treatment by AZD-6244, dasatinib and their combination. The *P* values for the comparisons between the drug combination and each individual drug are shown on the upper right couner. (**B**) The dose reduction index for the AZD-6244 and dasatinib combination as a function of percentage inhibition.

While inhibition by AZD-6244 and BMS-754807 seems to be a common feature of CRC cells, LS-174T, NCI-H747 and SK-CO-1 displayed sensitivity to dasatinib (Table 3). Interestingly, LS-174T was not sensitive to BMS-754807, but was sensitive to dasatinib. As shown in Figure 3, for some reason yet to be defined, BMS-754807 treatment did not result in an inhibition of AKT function in this cell line even though BMS-754807 did inhibit Akt activation in both LS-1034 and LS-153. The data in Figure 3 further indicated that dasatinib and AZD-6244 respectively inhibited Src function and Mek function. These results suggest that the MAP kinase pathway and Src kinases are independently contributing to the proliferation of LS-174T. We tested if AZD-6244 and dasatinib would have a synergistic effect on LS-174T proliferation (Figure 6). The dasatinib and AZD-6244 combination was indeed far more effective in blocking the proliferation of LS-174T than either drug alone. AZD-6244 and dasatinib had IC_50_’s of 1.41 μM and 0.61 μM, respectively, but the two drugs combined had an IC_50_ of 102 nM. The DRI value also increased dramatically with the increase in the percentage of inhibition, reaching 17-fold at 70% inhibition. The benefit was even higher at 80% inhibition, however, the DRI could not be calculated because AZD-6244 did not reach 80% inhibition up to 20 μM. These results are consistent with the mechanistic understanding that both Src kinase activity and the MAP kinase pathway independently contribute to the proliferation of LS-174T, and further suggest that a cancer based on this mechanism would be subject to combination targeted therapy with dasatinib and AZD-6244.

### 2.7. The Triple Combination of AZD-6244, BMS-754807 and Dasatinib Is Potently and Broadly Effective in Blocking CRC Cell Proliferation

**Figure 7:**
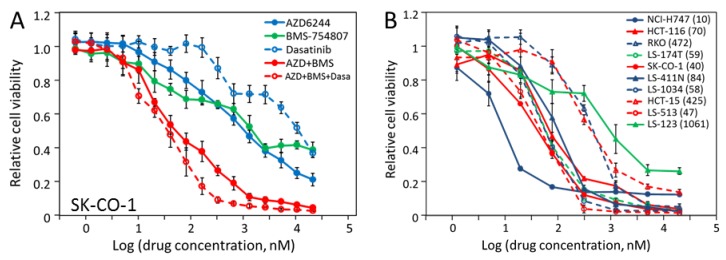
Inhibition of CRC cell proliferation by the triple combination of AZD-6244, BMS-754807 and dasatinib. (**A**) Response of SK-CO-1 to various drugs and drug combinations. The *P* values for comparing the AZD+BMS combination with either AZD-6244 or BMS-754807 are reported in Figure 4A. The *P* value for comparing the AZD+BMS+dasatinib triple combination to AZD+BMS double combination is 0.0066. (**B**) The inhibition of the proliferation of all CRC cell lines by the triple combination of AZD-6244, BMS-754807 and dasatinib (1:1:1 ratio) was determined as described in Materials and Methods. The IC_50_ (in nM) of the triple combination against each cell line is given in the parentheses following the name of the cell line.

Based on the results in the previous sections, most of the CRC cell lines were inhibited by AZD-6244, BMS-754807 and/or dasatinib, and two cell lines, SK-CO-1 and NCI-H747 were inhibited by all three. The synergistic effects in pair-wise combinations led us to determine if the three-drug combination would be even more potent than the two-drug combinations. To test this proposition, we tested SK-CO-1 against the 1:1:1 mixture of the three drugs at a series of concentrations (Figure 7A). The triple combination had an IC_50_ of 40 nM, marginally better than that of the AZD-6244 and BMS-754807 combination, at 52 nM. This could be due to the fact the dasatinib was a much weaker inhibitor than AZD-6244 and BMS-754807 for this cell line.

Although different cell lines were more potently inhibited by one combination or another, we thought that the triple combination may be a one-size-fits-all formulation for most CRC cells (except those that do not respond at all). We tested all 10 cell lines against the triple combination (1:1:1 ratio) (Figure 7B). The cells displayed IC_50_s ranging from 10 nM for NCI-H747 to 1061 nM for LS-123, with seven of the 10 cell lines inhibited by the triple combination with IC_50_’s below 84 nM. The extremely low IC_50_ of 10 nM for NCI-H747 is consistent with the observation that it is more sensitive to each drug alone than the other cell lines (Table 3). Interestingly, HCT-116 was not particularly sensitive to any of the three drugs individually, but had an IC_50_ of 70 nM for the triple combination. Three cell lines, RKO, HCT-15, and LS-123, that were not sensitive to inhibition by the individual drugs, had IC_50_’s of 425-1061 nM for the triple combination. If these 10 cell lines are representative of the spectrum of CRC molecular pathology, the triple combination is likely an excellent treatment for CRC in both potency and percentage of response. The IC_50_ values below 84 nM compare favorably to the IC_50_ values (80-600 nM) of imatinib inhibiting BCR-Abl dependent CML cell lines [38]. Further investigations in animal models and patients are needed to assess the efficacy of the triple combination as a CRC treatment. To make comparison more convenient, the IC_50_’s of the double and triple combinations are also shown to Table 3.

## 3. Discussion

### 3.1. Screening with Kinase Inhibitors Identify Potential Molecular Targets for Blocking CRC Proliferation

The molecular mechanisms driving the proliferation of CRC cells have been extensively studied [16,39]. The research identified KRAS/BRAF/Mek/Erk MAP kinase pathway activation as a driver pathway sustaining proliferation in many CRC cells. Other signaling pathways have also been reported to play important roles in some colorectal cancer cells [3,4]. However, the findings have not been translated into targeted therapy. In this study, we surveyed 10 CRC cell lines for their responses to a panel of kinase inhibitors individually or in combination. The following are the major findings. First, four of the 10 cell lines did not respond to any of the kinase inhibitors, and three of the four cell lines contain hypermutated genomes. Second, consistent with the mutational activation of the KRAS or BRAF and thus the MAP kinase signaling pathway, most cells are sensitive to the inhibitor of Mek kinase, AZD-6244. Further, most cells also displayed a common sensitivity to the IR/IGF-1R inhibitor BMS-754807, and some to the Src/Abl kinase inhibitor, dasatinib. Third, Western blotting analyses indicated that AZD-6244 inhibited the Erk activation by blocking Mek kinase activity, dasatinib blocked Src activation and function, and BMS-754807 blocks the activation of AKT activity and function, presumably through the inactivation of IR and/or IGF-1R. These results indicated that MAP kinase pathway, IR/IGF-1R-Akt pathway and/or Src kinases are all contributing to CRC cell proliferation, suggesting that these CRC cells are driven by several independent driver pathways. Finally, consistent with the multi-driver proliferation mechanism, the inhibition of CRC proliferation by each inhibitor was only moderate even for the most potent inhibitors, and combinations of the inhibitors are much more effective in blocking cell proliferation. These findings provide insights for understanding and treating CRC.

It is also noteworthy what kinase inhibitors did not inhibit the proliferation of the CRC cells. PTK inhibitors sunitinib, BGJ398, crizotinib, and imatinib did not inhibit any of the cell lines significantly, while gefitinib only inhibited one cell line, LS-513, with a modest IC_50_, suggesting that receptor PTKs in the PDGFR, EGFR, FGFR, MET, and soluble PTKs in the Abl families do not play major roles in driving the proliferation of CRC cells. This is largely consistent with the conclusion from genetic analysis that receptor PTKs are rarely mutated and are unlikely to play any major roles in driving CRC proliferation [16]. It is somewhat surprising that PI 3-kinase inhibitor AZD-6482 did not significantly inhibit any of the CRC cells, even though four of CRC cell lines contain mutations in PI 3-kinase. Indeed PI 3-kinase is one of the most frequently mutated genes in CRC, and it has been suggested that CRC cells are likely to respond to PI 3-kinase inhibitors [16]. The present study suggests that either the mutations do not activate the PI 3-kinase pathway, or the activated PI 3-kinase pathway does not play a significant role in CRC proliferation. EGFR has been a target for targeted therapy for CRC with limited success. The lack of inhibition of virtually all the CRC cell lines by gefitinib indicates that EGFR plays at most a minor role in CRC cell proliferation.

### 3.2. Hypermutation De-Sensitizes Cancer Cell Response to Kinase Inhibitors

Four of the cell lines did not respond to the individual kinase inhibitors, and interestingly three of them contained hypermutated genomes. At least two scenarios are plausible. Because these cells’ genomes contain 10–20 times as many mutations as other cancer cell lines, it is possible that many signaling pathways are affected. About 8% all cancers contain hypermutated genomes. A recent study determined that normal cancers contain an average of four driver mutations/tumor, and hypermutated genomes likely contain many times more [34]. It is plausible that in such hypermutated cancers, so many signaling pathways are activated, and given that signaling pathways often cross activate, blocking any one pathway simply has very little effect on the cell proliferation. It is also possible that certain drug resistance mechanisms, due to drug exclusion or drug metabolism, may be activated by some mutations. Whatever the mechanism is, these cancers are likely most resistant to targeted cancer therapy. 

### 3.3. CRC Cells Are Dependent on MAP Kinase Pathway, IR/IGF-1R/AKT, and/or Src Kinases for Proliferation

Despite the success of targeted therapy in treating some cancers, it has not reached most cancers. One of the most basic challenges targeted therapy faces is that it has worked effectively only for cancers that are addicted to a driver oncogene or pathway. Successful applications of Abl inhibitor for BCR-Abl-dependent chronic myeloid leukemia, EGFR inhibitors for EGFR mutation dependent non-small cell lung cancer, and ErbB2 inhibitors for ErbB2-dependnet breast cancer are all examples of both the power of targeted therapy and the challenge it faces. However, most cancers contain multiple driver genes that may independently contribute to the proliferative advantage of a cancer cell. A good strategy for dealing with this has not been worked out. Most of the CRC cells we studied here appear to be multi-driver cancer cells. None of the 10 CRC cell lines displayed an exclusive reliance on any one gene or pathway, as most of them contain activated MAP kinase, IR/IGF-1R/AKT, and/or the Src kinase pathways.

Even though there is a certain level of heterogeneity among the tested cells, the level of similarity is also surprising. While it is not surprising that all six sensitive cells responded to AZD-6244, due to the mutation of BRAF and KRAS in these cells, it was surprising that five of the six sensitive cells responded to BMS-754807, because none of these cells contains a mutation in IR or IGF-1R. However, the relationship between IR/IGF-1R signaling is complicated by cros-talks and feedback loops [40,41,42,43]. IR and IGF-1R can activate both the MAP kinase pathway and the PI 3-kinase pathway, yet in the CRC cells, inhibition of IR and IGF-1R primarily blocked Akt activation, and reduced Akt function. This suggested the main pathway under the regulation of IR and IGF-1R is the PI 3-kinase pathway. Also supporting this conclusion is the exception to this relationship, LS-174T, the only sensitive cell that was not inhibited by BMS-754807. Western blot analysis indicates that BMS-754807 did not block the activation of AKT in this cell (Figure 3). It is unclear how in most of these cells, IR/IGF-1R become a driver of proliferation, and why LS-174T becomes an exception to this regulatory relationship. 

The inhibition of several cell lines by dasatinib suggests that Src is playing a role in driving the proliferation of these cells. Western blot analyses confirmed that dasatinib indeed blocked Src activation by autophosphorylation, and thus Src kinase activity. Indeed Src has been reported to be associated with some CRC cell lines [44,45,46]. These analyses together demonstrate that CRC cell proliferation is driven by the MAP kinase pathway, IR/IGF-1R/AKT pathway, and/or Src kinase signaling.

### 3.4. A Rationale for Combination Therapy Using Multiple Signaling Inhibitors

The multi-driver nature of CRC cell proliferation could explain why CRC cannot be effectively treated by individual kinase inhibitors, even though these inhibitors clearly block their intended targets, and their intended targets clearly play important roles in CRC cell proliferation. The multi-driver nature of CRC cells also dictates that effective targeted therapy against CRC would require the combination of several inhibitors, each blocking one activated pathway. Our results confirmed this hypothesis. If the multi-driver mechanism applies to other solid tumors, then the combination of kinase inhibitors may be an effective and necessary approach of targeted therapy. With the realization of the limits of mono-agent targeted therapy [47,48], combination targeted therapy has attracted significant attention [49]. However, drug combinations are often limited by lack of efficacy and dose limiting toxicity [49]. Finding strategies to lower the needed dose to achieve a desired level of inhibitory effect is the key to the future success of this new approach.

A key feature of the AZD-6244/BMS-754807 or AZD-6244/dasatinib drug combination is that the synergy increases dramatically with the level of inhibition. While the synergy at 10–20% inhibition is often minimal, it increases dramatically as the level of inhibition increases. At 60–70% inhibition, the dose reduction index often reached higher than 10, and as high as more than 30. This feature is fully consistent with the multi-driver proliferation mechanism, and would be particularly beneficial for combination targeted therapy. To inhibit the proliferation of these cells to a significant level, for example 70–80%, with an individual drug would require concentrations of 20 μM or higher even for the most potent inhibitors. Signaling inhibitors at such high concentrations would likely cause off-target effects and significant toxicity [50,51]. This consideration could explain why single agent targeted therapy is unlikely to work for multi-driver cancers, such as CRC. However, with combination targeted therapy, 70–90% inhibition can often be reached by combinations of two or three signaling drugs, each at nM levels. At the nM concentrations, the kinase inhibitors are much more specific, and would likely cause minimal general toxicity.

### 3.5. A Triple Combination for CRC Targeted Therapy

The study demonstrated that double or triple combinations of AZD-6244, BMS-754807 and dasatinib are particularly effective in blocking the proliferation of most CRC cell lines, with many IC_50_ values below 100 nM. As a benchmark for targeted therapy, CML cells dependent on BCR-Abl for proliferation are inhibited by Abl inhibitor imatinib with IC_50_ values in the range of 80–600 nM (38). Projecting these results from this study into patient treatment provides at least two treatment approaches that are worthy of further investigation. First, if a patient can be pre-tested for the drug sensitivity to decide which drug combination the patient would respond to, then an effective drug combination can be selected for the patient. This is the ideal scenario of targeted therapy or precision therapy. Second, if a drug sensitivity pre-test is not feasible, the triple combination of AZD-6244, BMS-754807 and dasatinib may be an attractive treatment option because majority of CRC cell lines can be strongly inhibited by the combination (IC_50_ ≤ 84 nM). The treatment with a triple combination may have another benefit. Increasing evidence demonstrates significant intra-tumor genetic heterogeneity, where a solid tumor may contain heterogeneous sub-population of cancer cells driven by different genetic mutations [52,53]. If the heterogeneity within CRC tumor samples can be represented by the cell lines in our panel, then the triple combination should block the proliferation of most of the different sub-populations. 

## 4. Materials and Methods

### 4.1. Cell Lines, Media and Drugs

All CRC cell lines were purchased from ATCC (Manassas, VA, USA), and were used within 6 months of their purchase. ATCC authenticates all its cell lines by short tandem repeat profiling, cell morphology monitoring, karyotyping, and cytochrome C oxidase I testing. The cells were grown in ATCC-formulated McCoy’s 5a Medium Modified (Catalog No. 30-2007), RPMI-1640 Medium (Catalog No. 30-2001), or Eagle’s Minimum Essential Medium (Catalog No. 30-2003), containing 10% FBS and 1% penicillin-streptomycin (Thermo Fisher Scientific, Waltham, MA, USA). All cells were cultured at 37 °C in humid atmosphere containing 5% CO_2_. Kinase inhibitors were purchased from Selleckchem (Munich, Germany), LC Laboratories (Woburn, MA, USA) or AdooQ Bioscience (Irvine, CA, USA). 

### 4.2. Cell Culture and Viability Assays

Cells were plated into 96-well plates at 25,000 cells per well, and were incubated with drugs at indicated concentrations in 100 μL medium containing 1% DMSO for 72 h. For adherent cells (all cells used except NCI-H747), at the end of the incubation, 10 μL of staining solution containing 5 mg/mL MTT dye (3-(4,5-dimethylthiazol-2-yl)-2,5-diphenyltetrazolium bromide) (Thermo Fisher Scientific) was added into each well. After incubation at 37 °C for another 4 h, the medium in the wells was removed by aspiration. DMSO (100 μL) was added into each well, and when purple formazon was dissolved, absorbance at 490 and 750 nm for each well was determined using a VersaMax Tunable Microplate Reader with SOFTmax Pro v5 (Molecular Devices, Sunnyville, CA, USA). The A490-A750 values were taken as indicator of cell viability. For semi-suspension cells (NCI-H747), cell viability was determined by staining the cells with Biolog Redox Dye Mix MB (Biolog Inc. Hayward, CA, USA). At the end of 72 h drug treatment, 10 μL Biolog Redox Dye Mix MB was added into each well, and the plates were incubated at 37 °C for 2 h before the absorbance of the water soluble formazan product at 590 and 750 nm was determined using the microplate reader. The A590-A750 values were taken as indicator of cell viability. All cell growth and drug inhibition experiments were performed in triplicates at least twice. Statistical analysis was performed on six repeat data sets for each assay.

### 4.3. Initial Four-Concentration Screening and IC_50_ Determination

To determine if the proliferation of a cell line is sensitive to inhibition by the kinase inhibitors, each cell was screened against a panel of ten kinase inhibitors (Table 1) at four concentrations, 10 nM, 100 nM, 1 μM, and 10 μM. Cells treated at identical conditions without the inhibitors were used as controls. Relative viability was calculated relative to the control as 1. If the proliferation of a cell line was inhibited more than 50% at 10 μM, the IC_50_ of the cell line for the drug was also determined using a 16-concentration 2x dilution series of the drug, ranging from 0.6 nM to 20 μM. The IC_50_ values were estimated from the dose response curves as the concentrations that resulted in 50% inhibition of the cell’s viability. 

### 4.4. Western Blot Analysis of Drug Effects on Cell Signaling

To determine the effects of the kinase inhibitors on the signaling network in the CRC cell lines, the cell lines were cultured to 70% confluency, and then treated by the indicated drugs. The culture medium was then replaced by fresh medium containing kinase inhibitors at designated concentrations. The cells were incubated for 2 h under treatment. After the treatment, the culture medium was aspirated, and the cells were then resuspended in the SDS-PAGE loading buffer containing a protease inhibitor cocktail for mammalian cell and tissue extracts (Sigma-Aldrich, St. Louis, MO, USA) and protein phosphatase inhibitors (PhosSTOP, Sigmal-Aldrich) and lysed in a 95 °C heat bath immediately. The lysates were then cleared by centrifugation and the supernatants were fractionated by SDS-PAGE, and immunoblotted by various antibodies recognizing selected signaling proteins and their specific phosphorylated forms. The loading was normalized based on the β-actin expression levels in these lysates determined by an antibody specific for this protein. All antibodies were purchased from Cell Signaling Technology (Danvers, MA, USA).

### 4.5. Drug Synergy Analysis and Combination Index Calculation

To determine if two drugs were synergistic in their inhibition of a cell line’s proliferation, the cell line viability was determined by incubating the cells in the presence of each drug alone or both drugs (1:1 ratio) at 16 concentrations ranging from 0.6 nM to 20 μM. The concentration of a drug or drug combination that inhibited cell proliferation at X% (IC_x_) was determined manually from the graphs of drug dose response curves. The combination index (CI) was calculated for a given percentage of viability inhibition according to Chou [36], using the Chou and Talalay Method [37]. Briefly, the IC_40_, IC_50_, IC_60_, etc., were determined from the dose response curves, and the CI at a given level of inhibition (X%) was calculated by the following equation: CI = IC_x_ab/IC_x_a + IC_x_ab/IC_x_b, where IC_x_ab is the concentration of the drug combination that resulted in X% inhibition, and IC_x_a and IC_x_b are the concentrations of drug a and drug b, respectively, that caused X% inhibition to cell proliferation. The dose reduction index (DRI), a measure of how many folds the combination concentration may be reduced, for a given percentage of inhibition, compared with the doses of each drug alone, is calculated as: DRI = 1/CI [37].

### 4.6. Statistical Analysis

We performed three types of statistical analysis to evaluate the data. First, the IC_50_’s are reported as the mean standard deviation, which were determined from at least two repeat assays, each in triplicates. Thus each IC_50_ and the associated standard deviation were calculated from six IC_50_’s determined from six graphs. Second, when the potency of a two-drug combination was compared to an individual drug, the *P* value was calculated comparing two sets of inhibition data at 16 drug concentrations. The *P* value was calculated using the TTEST function in Excel with the assumption of two tailed distribution and as a paired t-test. Third, when the IC_50_ values are compared between a drug combination and an individual drug, the mean and the standard deviation were calculated from six IC_50_ values. To compare the IC_50_’s from two different drug treatments, the *P* value was calculated comparing two sets of IC_50_ values (each set contains six IC_50_’s) using Excel with the assumption of two tailed distribution and equal variance. The *P* values are reported in the appropriate figure legends.

### 4.7. Mutation Information of CRC Cell Lines

Genomic data for the CRC cell lines were collected from the COSMIC database, the Catalogue of Somatic Mutations in Cancer (https://cancer.sanger.ac.uk/cosmic) [25].

## Figures and Tables

**Table 1:** Kinase inhibitors used in this study and the target cancer census protein tyrosine kinase they inhibit. The inhibitors below, with the exception of BGJ398 and BMS-754807, have been tested against majority of the kinases in the kinome (http://lincs.hms.harvard.edu/kinomescan/). The target cancer census kinases with *K_d_* values below 20 nM are listed as the number in the parenthesis in nM. BGJ398 and BMS-754807 have not been tested against the kinome. The data for BGJ398 and BMS-754807 are taken from references 23 and 21, respectively. The IC_50_ of AZD-6244 against MEK 1 is taken from reference 24.

Inhibitor	Target Kinases (*K_d_* in nM)
BGJ398	FGFR1 (0.9), FGFR2 (1.4), FGFR3 (1), FGFR4 (60)
BMS-754807	IGF1-R (1.8), insulin receptor (1.7), Met (5.6)
Bosutinib	Abl1 (0.12), Abl2 (1.5), EGFR3 (0.77), EphA3 (5.8), Lck (0.59), Src (1), BTK (4.8)
Crizotinib	Alk (3.3), Met (2.1)
Dasatinib	Abl1 (0.03) Abl2 (0.17), PTK6 (7.8), EGFR3 (18), EphA3 (0.09), CSF1R (0.58), Kit (0.81), PDGFRA (0.47), PDGFRB (0.63), Lck (0.2), Src (0.21), Btk (1.4)
Gefitinib Imatinib	EGFR (1) Abl1 (1.1), Abl2 (10), CSF1R (11), Kit (13), PDGFRB (14)
Sunitinib	CSF1R (2.5), FLT3 (0.4), Kit (0.4), PDGFRA (0.8), PDGFRB (0.1), Ret (13), ITK (13), VEGFR2 (1.5)
AZD-6244	Mek 1 (14)
AZD-6482	PIK3C (0.69)

**Table 2:** CRC cell lines studied in this report and their characteristics.

Cell Line	Pik3ca Mutations	Braf Mutations	KRAS Mutations	Cancer Census Mutations	Coding Mutations
HCT-15	E545K, D549N	none	G13D	321	11348
HCT-116	H1047R	none	G13D	163	4318
LS-1034	None	none	A146T	10	207
LS-123	None	none	G12S	13	483
LS-411N	None	V600E	none	209	7141
LS-174T	H1047R	D211G	G12D	127	3517
LS-513	None	none	G12D	21	444
NCI-H747	None	none	G13D	14	377
RKO	H1047R	V600E	none	155	4762
SK-CO-1	None	none	G12V	16	443

**Table 3:** IC_50_ of signaling small molecule drugs inhibiting the proliferation of CRC cell lines. ^a^ Combination between AZD-6244 and BMS-754807; ^b^ Combination between AZD-6244 and dasatinib; ^c^ ND, not determined; ^d^ ABD Tripo: triple combination of AZD-6244, BMS-754807 and dasatinib.

Cell	AZD-6244	BMS-754807	Dasatinib	Gefitinib	Bi-Combo	ABD Tripo ^d^
LS-1034	1.09 ± 0.16	0.42 ± 0.13	>10	>10	0.153 ± 0.027 ^a^	0.058 ± 0.005
LS-174T	1.41 ± 0.63	>10	0.61 ± 0.11	>10	0.102 ± 000 ^b^	0.059 ± 0.006
LS-411N	3.01 ± 0.62	5.07 ± 1.24	>10	>10	0.146 ± 0.088 ^a^	0.084 ± 0.028
LS-513	0.95 ± 0.21	0.42 ± 0.27	>10	0.94 ± 0.44	0.086 ± 0.030 ^a^	0.047 ± 0.040
NCI-H747	0.28 ± 0.05	1.39 ± 1.02	0.26 ± 0.12	>10	0.046 ± 0.005 ^a^	0.010 ± 0.001
SK-CO-1	1.15 ± 0.34	1.43 ± 0.33	10.4 ± 1.67	>10	0.052 ± 0.021 ^a^	0.040 ± 0.004
HCT-116	>10	>10	>10	>10	ND ^c^	0.070 ± 0.005
HCT-15	>10	>10	>10	>10	ND	0.425 ± 0.068
LS-123	>10	>10	>10	>10	ND	1.061 ± 0.388
RKO	>10	>10	>10	>10	ND	0.472 ± 0.025

## Abbreviations

CI	combination index
CRC DRI	colorectal cancer dose reduction index
IGF-1R	insulin-like growth factor 1 receptor
IR	insulin receptor
MAP kinase	mitogen activated protein kinase
PI 3-kinase	phosphatidylinositol 3-kinase
PTK	receptor protein tyrosine kinase
